# Chemical Composition of the *Cinnamomum malabatrum* Leaf Essential Oil and Analysis of Its Antioxidant, Enzyme Inhibitory and Antibacterial Activities

**DOI:** 10.3390/antibiotics12050940

**Published:** 2023-05-22

**Authors:** Aswathi Moothakoottil Kuttithodi, Arunaksharan Narayanankutty, Naduvilthara U. Visakh, Joice Tom Job, Berin Pathrose, Opeyemi Joshua Olatunji, Ahmed Alfarhan, Varsha Ramesh

**Affiliations:** 1 Division of Cell and Molecular Biology, PG & Research Department of Zoology, St. Joseph’s College (Autonomous), Devagiri, Calicut 673008, Kerala, India; 2 Department of Agricultural Entomology, College of Agriculture, Kerala Agricultural University, Thrissur 680656, Kerala, India; 3 African Genome Center, Mohammed VI Polytechnic University, Ben Guerir 43150, Morocco; 4 Traditional Thai Medical Research and Innovation Center, Faculty of Traditional Thai Medicine, Prince of Songkla University, Hat Yai 90110, Thailand; 5 Department of Botany and Microbiology, College of Science, King Saud University, P.O. Box 2455, Riyadh 11451, Saudi Arabia; 6 Department of Biotechnology, Deakin University, Geelong, VIC 3217, Australia

**Keywords:** *Cinnamomum malabatrum*, GC-MS analysis, essential oil, antioxidant activity, antibacterial activity, enzyme inhibitory activity

## Abstract

*Cinnamomum* species are a group of plants belonging to the Lauraceae family. These plants are predominantly used as spices in various food preparations and other culinary purposes. Furthermore, these plants are attributed to having cosmetic and pharmacological potential. *Cinnamomum malabatrum* (Burm. f.) J. Presl is an underexplored plant in the *Cinnamomum* genus. The present study evaluated the chemical composition by a GC-MS analysis and antioxidant properties of the essential oil from *C. malabatrum* (CMEO). Further, the pharmacological effects were determined as radical quenching, enzyme inhibition and antibacterial activity. The results of the GC-MS analysis indicated the presence of 38.26 % of linalool and 12.43% of caryophyllene in the essential oil. Furthermore, the benzyl benzoate (9.60%), eugenol (8.75%), cinnamaldehyde (7.01%) and humulene (5.32%) were also present in the essential oil. The antioxidant activity was indicated by radical quenching properties, ferric-reducing potential and lipid peroxidation inhibition ex vivo. Further, the enzyme-inhibitory potential was confirmed against the enzymes involved in diabetes and diabetic complications. The results also indicated the antibacterial activity of these essential oils against different Gram-positive and Gram-negative bacteria. The disc diffusion method and minimum inhibitory concentration analysis revealed a higher antibacterial potential for *C. malabatrum* essential oil. Overall, the results identified the predominant chemical compounds of *C. malabatrum* essential oil and its biological and pharmacological effects.

## 1. Introduction

Spices are an important class of plants and have been traditionally used all over the world. The different plant species belonging to the *Zingiberaceae*, *Lauraceae*, *Myrtaceae* and *Schisandraceae* families are reputed spice products [1,2]. The source of spices includes the leaves, roots, stem bark, buds and flowers [3]. The spices are predominantly used as dietary ingredients or supplements that enable the flavoring of the cuisines [4,5]. The spices are traditionally used in medicinal systems including Ayurveda and Chinese medicines for the management of various illnesses [6,7]. In addition, the plants are well-known for their cosmetic potential as well as pharmaceutical activities. The prevention of infectious diseases by controlling the microbial communities and multidrug resistance is an integral function of the spices. In addition, the novel “spiceceuticals”, the pharmacologically active spice products, are also emerging against numerous degenerative disorders including metabolic syndromes and cancers [8,9].

The *Cinnamomum* spp. are important spices that are used for various purposes in different parts of the world. The genus *Cinnamomum* comprises approximately 250 species that are distributed in the Asian and Australian continents [10]. Among these, the *C. zeylanicum* and *C. cassia* are the prominent representatives of the genus *Cinnamomum*. The *C. zeylanicum* (now known as *C. verum*) is the “true cinnamon”, which is also known as “Ceylon cinnamon” [11]; the *C. cassia* (previously *C. aromaticum*) is known as “Chinese cinnamon” [12]. The most important cinnamon oils in the world trade are those from *Cinnamomum zeylanicum* (or *C. verum*), *C. cassia* and *C. camphora* [13]. Among the different plants, *C. zeylanicum* is well-studied; the antibacterial properties are also attributed to *C. zeylanicum* leaves and their bioactive compounds against clinically drug-resistant bacteria [14]. Further, a study by Assaran et al. [15] indicated the protective effect of *C. zeylanicum* extract on pentylenetetrazole-induced seizure. It was also effective in preventing the doxorubicin-mediated oxidative damage to the heart tissue and subsequent cardiomyopathy [16]. The bark extract of the plant was protective against gentamicin-induced renal toxicity by preventing inflammatory insults [17]. It was also found to protect against formaldehyde-mediated inflammation and apoptosis in neurons [18]. The extracts of *C. zeylanicum* were also known to have antidepressant properties in murine models [19]. Furthermore, the anticancer activities were also evident for *C. zeylanicum* extract by modulating various cellular signaling cascades [20]. *C. zeylanicum* extract was also an effective antimicrobial agent against the infection of *Toxoplasma gondii* in murine models [21]. The *C. cassia* is another important plant belonging to the family; it was effective against the gastrointestinal toxicities in animal models [22]. *C. burmannii* is also attributed to having pharmacological effects; the administration of the extract improved hepatic redox balance and subsequently protected against high-fat diet-mediated liver toxicity [23]. *C. burmanii* was also effective against bacterial pathogens by inhibiting bacterial proliferation and blocking biofilm formation [24].

The essential oils isolated from different *Cinnamomum* spp. are another important extract with potential insecticidal and pharmacological properties. The *C. camphora* essential oil was found to be effective against bacterial forms and dust mites [25]. The essential oil was also effective against bacterial strains that are antibiotic-resistant [26]. It was also found to be useful in the management of mosquitoes by killing the larval forms of *Anopheles stephensi* [27]. Besides their insecticidal and antimicrobial properties, pharmacological properties are also attributed to *C. camphora* essential oil. The essential oil had anti-inflammatory properties in cultured cells and animal models [28,29]. Further, the essential oil had analgesic properties in animal models [30]. The essential oil was also effective in preventing learning impairment and memory loss in mice [31]. Apart from the plant, *C. burmannii* was shown to have radical quenching anticancer properties [32]. The *C. zeylanicum* essential oil was also shown to have antibacterial and antineoplastic properties [33]. Likewise, the essential oil of *C. verum* was reported to have protective efficacy against CCl4-induced hepato-renal toxicities in rats [34]. The essential oil-based nanoemulsions of *C. litseifolium* were shown to have antioxidant and hypoglycemic activities [35]. The *C. glanduliferum* essential oil was shown to protect against ethanol-induced inflammation and gastritis in rats [36]. The essential oil of *C. osmophloeum* was reported to have lipid-lowering properties in mice, and the efficacy was comparable to the bioactive compounds such as linalool [37]. The essential oil was also found to be effective against pancreas toxicity [38] and endotoxin-induced intestinal damage [39].

Among the different species of the *Cinnamomum* genus, the *C. malabatrum* (Burm.f.) J.Presl is an endemic medicinal plant that belongs to the Western Ghats, Kerala, India. Limited studies are available on the essential oil of the plant; Leela et al. [40] indicated the chemical composition of the essential oil of *C. malabatrum* where (E)-Caryophyllene (28.6%), (E)-Cinnamyl acetate (15.1%) and Bicyclogermacrene (14.4%) were the predominant compounds. Later, a study by Sriramavaratharajan and Murugan [41] reported the predominant compounds as β-Phellandrene (12.0%) and linalool (15.4%). The present study, therefore, aimed to extract the essential oil from the *C. malabatrum* leaves and analyze its chemical composition. Further, the radical quenching properties of the essential oil and its enzyme-inhibitory properties were evaluated using in vitro models. The enzyme-inhibition activity was assessed in terms of diabetes-associated enzymes; the α-amylase and α-glucosidase are major enzymes associated with carbohydrate metabolism and thereby contribute to type 2 diabetes mellitus [42,43], and are a prominent target for antidiabetic drugs [44,45]. The activation of polyol pathway enzymes aldose reductase and sorbitol dehydrogenase plays a crucial role in the microvascular complications of diabetes [46,47,48]. The antibacterial activity was also determined using two methods: the disc diffusion method and minimum inhibitory concentrations.

## 2. Results

### 2.1. C. malabatrum Essential Oil Yield and Chemical Contents

**Figure 1:**
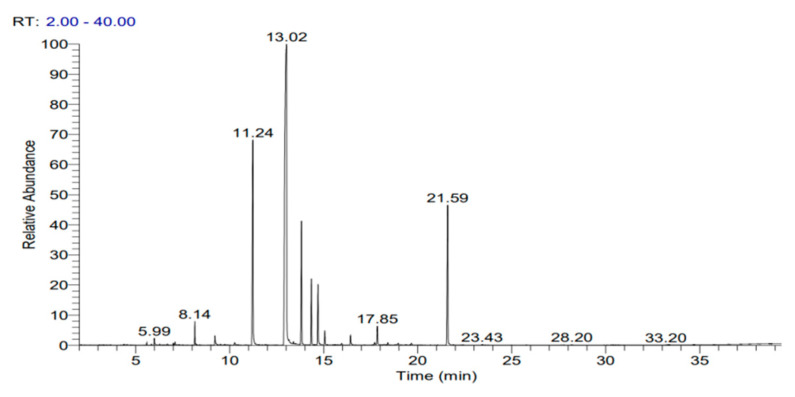
The chromatograms of GC–MS analysis of *C. malabatrum* leaf essential oil.

The yield of *C. malabatrum* leaf essential oil was 0.72 ± 0.13% using the hydro-distillation method. The GC-MS chromatogram of the essential oil is shown in Figure 1. There were eleven main peaks observed in the chromatogram.

The results of the GC-MS analysis indicated the presence of 38.26 ± 0.41% of linalool, 12.01 ± 0.54% of cinnamaldehyde and 11.43 ± 0.52% of caryophyllene in the essential oil. In addition, the benzyl benzoate (9.60 ± 0.05%), eugenol (8.75 ± 0.23%) and humulene (5.32 ± 0.12%) were also present in the essential oil (Table 1).

### 2.2. Antioxidant Effects of C. malabatrum Essential Oil

We observed a dose-dependent scavenging of various free radicals in *C. malabatrum* essential oil treatments (Table 2). The IC_50_ values were found to be less than 100 µg/mL in the entire radical quenching assay for the essential oil. Further, among the different radicals analyzed, the DPPH was more sensitive to the essential oil treatment. However, the radical quenching properties of the CMEO were significantly lower than those of ascorbic acid (*p* < 0.001). On the contrary, the CMEO was having a higher antioxidant potential in terms of DPPH and ABTS radical scavenging (*p* < 0.001). The peroxide scavenging and lipid peroxidation potential of the linalool were higher than the CMEO (*p* < 0.001). The reducing potential (FRAP) of *C. malabatrum* essential oil was significantly lower than the ascorbic acid, whereas it was significantly higher than the linalool (*p* < 0.001).

### 2.3. Enzyme-Inhibitory Activities of C. malabatrum Leaf Essential Oil

The enzyme-inhibitory activities of the essential oil were evaluated using different enzymes associated with diabetes and diabetic complications. The *C. malabatrum* was found to inhibit the enzymes such as α-amylase and α-glucosidase (Table 3); however, the bioactive compounds linalool and ascorbic acid were found to be more potent inhibitors of these enzymes. The inhibition of aldose reductase and sorbitol dehydrogenase was also observed in CMEO treatment with the respective IC_50_ values 82.90 ± 0.67 and 98.61 ± 3.18 µg/mL. However, a more significant inhibition in the ascorbic acid treatment 28.70 ± 2.14 and 60.09 ± 1.32 µg/mL (*p* < 0.001) was observed. Likewise, the linalool also showed significant inhibition but to a lesser extent than the ascorbic acid (*p* < 0.001).

### 2.4. Antibacterial Effects of C. malabatrum Essential Oil

The antibacterial potential of the *C. malabatrum* essential oil was tested against both Gram-positive and Gram-negative organisms using the disc diffusion method (Table 4), and also in terms of minimum inhibitory concentration (Table 5). The antibacterial activity was found to be similar in CMEO to that of GM in the *Pseudomonas aeruginosa* strain (*p* = 0.3150). Likewise, the CMEO was effective as that of linalool against *Escherichia coli* and *Salmonella enterica*. However, in other strains, a significantly higher antibacterial activity was observed for CMEO than linalool.

The minimum inhibitory concentrations of CMEO were comparable for the *C. malabatrum* essential oil and linalool in the *Bacillus cereus* (*p* = 0.0028). Likewise, the MIC values of linalool and GM were similar in *P. aeruginosa*, *S. aureus*, *S. pyogenes* and *S. enterica* (*p* > 0.05). The antibacterial activity of CMEO was significantly lower than that of gentamicin (*p* < 0.001).

## 3. Discussion

*Cinnamomum* spp. is well-known for its culinary uses in different parts of the world. In addition, the essential oil extracted from the spice is of cosmetic and pharmacological uses. Among these, the *C. verum*, *C. zeylanicum* and *C. tamala* are widely evaluated. The *C. malabatrum* is an endemic plant which is less explored for its biological and pharmacological properties. The present study evaluated the chemical components of the plant essential oil by a GC-MS analysis.

The results of the GC-MS analysis indicated the presence of 38.26% of linalool, 12.01% of cinnamaldehyde and 11.43% of caryophyllene in the essential oil. Furthermore, the benzyl benzoate (9.60%), eugenol (8.75%) and humulene (5.32%) were also present in the essential oil. A previous study by Leela, Vipin, Shafeekh, Priyanka and Rema [40] indicated that (E)-Caryophyllene (28.6%), (E)-Cinnamyl acetate (15.1%) and Bicyclogermacrene (14.4%) were the predominant compounds. Further, Benzyl benzoate (8.5%), α-Humulene (4.7%), Globulol (2.7%) and β-Phellandrene (2.2%) were other minor compounds present in the leaf essential oil according to their study. On the contrary, another study by Sriramavaratharajan and Murugan [41] indicated the presence of β-Phellandrene (3.5–12.0%), linalool (13.1–15.4%), (E)-Caryophyllene 8.4–31.4%) and Bicyclogermacrene (12.9–20.0 %) in the essential oil.

The dose-dependent scavenging of various free radicals in *C. malabatrum* essential oil treatments was observed. The IC_50_ values were found to be less than 100 µg/mL in the entire radical quenching assay for the essential oil. Further, among the different radicals analyzed, the DPPH was more sensitive to the CMEO treatment. In addition, it was interesting to note that the ferric-reducing potential of the CMEO was comparable to that of the standard ascorbic acid. The free radicals are important agents associated with oxidative stress and inflammation [49]. Hence, the radical quenching is important to prevent the oxidative damage to cellular macromolecules, and thereby prevent various degenerative diseases [50,51]. Hence, the inhibition of the radicals by *C. malabatrum* indicates the potential of the essential oil in preventing chronic diseases. Further, the compounds such as linalool [52], caryophyllene [53] and cinnamaldehyde [54,55] are shown to prevent oxidative damage in various conditions of animal models and clinical studies. Hence, it must be possible that the bioactive stress volatiles of the *C. malabatrum* might be responsible for the antioxidant potentials.

The enzyme inhibitory activities of the essential oil were evaluated using different enzymes associated with diabetes and diabetic complications. The *C. malabatrum* was found to inhibit the enzymes such as α-amylase and α-glucosidase. The α-amylase and α-glucosidase are two enzymes associated with type 2 diabetes mellitus [42,43]. Several synthetic drugs are known to inhibit these enzymes as a preventive measure to diabetes, and thereby making these enzymes an antidiabetic drug target [44,45]. Likewise, the secondary diabetic complications including retinopathy, nephropathy and cardiomyopathy are another important concern of diabetic patients [56,57]. The activation of polyol pathway enzymes aldose reductase and sorbitol dehydrogenase plays a crucial role in the microvascular complications of diabetes [46,47,48]. Numerous plant products and bioactives are reported to interfere with polyol enzymes and are thereby found to be protective against the microvascular complications of diabetes [58,59]. Hence, the *C. malabatrum* essential oil may prove beneficial against diabetes and associated microvascular complications.

The antibacterial potential of the *C. malabatrum* essential oil was tested against both Gram-positive and Gram-negative organisms using the disc diffusion method, and also in terms of minimum inhibitory concentration. The selected microorganisms are known to be associated with various diseases in humans, animals and poultry. The *E. coli* is reported to cause infections in urinary and respiratory tracts [60]; whereas, the *P. aeruginosa* is associated with wound infections during surgery and transplantations [61]. *Staphylococcus* and *Streptococcus* are associated with cutaneous and genital infections in humans causing various diseases [62,63]. The *Bacillus cereus* is an important pathogen which is known to produce toxins and is subsequently associated with food poisonings, and it is often fatal [64,65]. Hence, the inhibition of the growth of these organisms by the *C. malabatrum* essential oil may be indicative of its antibacterial potential. Further, the bioactive compounds present in the essential oil such as linalool, caryophyllene and cinnamaldehyde are known for their antimicrobial properties [66,67,68]. It is therefore possible that the antibacterial properties exhibited by the *C. malabatrum* essential oil may be attributed to the bioactive metabolites present in it. Hence, the present study confirms the chemical components as well as the antibacterial and antidiabetic properties of the leaf essential oil of *C. malabatrum*.

## 4. Materials and Methods

### 4.1. Collection of C. malabatrum Leaves and Extraction of Essential Oil

The leaves of *Cinnamomum malabatrum* (voucher specimen number KFRI-26/2020 was deposited in KFRI, Peechi, India) were obtained from the cultivation area of Kerala Agricultural University (10.54544° N, 76.28830° E), Thrissur, India. The extraction of the essential oil was by a Clevenger-type apparatus using the hydro-distillation method. The essential oil was dehydrated using sodium sulfate (anhydrous). and stored in the dark during cooling.

### 4.2. Chemical Component Analysis by GC-MS Analysis

The characterization of the essential oil extracted from *C. malabatrum* was carried out using the TSQ 8000 Evo GC-MS instrument (Thermo Scientific, Waltham, Massachusetts, USA) with an autosampling unit. The TG-5MS chromatographic column (30 mm × 0.25 mm × 0.25 μm) with helium (1 mL/min) as the carrier gas was used in the analysis. The oven temperature of the system was set at 50 °C with a ramp temperature of 10 °C/min until 120 °C; later, the ramp was 5 °C per minute, and finally fixed at 270 °C. The chemical composition was analyzed by the matching of MS spectra with the NIST library, and the retention index (RI) values were estimated by calibrating their instrument with a homologous series of alkenes (C_7_–C_30_ n-alkene) under the same conditions [69]. 

### 4.3. Antioxidant Activities of C. malabatrum Leaf Essential Oil

The concentration of essential oil used was a different series from 0 to 100 µg/mL for each radical quenching assay. The quenching of DPPH radicals was analyzed using the methods of [70]. The ABTS radicals scavenging activity was analyzed according to the methods of Li et al. [71]. The hydrogen peroxide quenching potential of the essential oil was following the methods of Munteanu and Apetrei [72]. The ferric-reducing abilities of the essential oil were estimated according to the methods described by He et al. [73]. The methods described by Okoh et al. [74] were followed for the lipid peroxidation inhibition assay.

### 4.4. Enzyme-Inhibitory Properties of C. malabatrum Leaf Essential Oil

The enzyme-inhibitory potential of the essential oil was evaluated by mixing different concentrations (0–100 μg/mL) against the respective enzymes and their substrates. The enzyme activities will be estimated in terms of the substrate utilized after incubation. The inhibition of α-amylase [75], α-glucosidase [76], aldose reductase [77] and sorbitol dehydrogenase [78] was carried out according to the methods previously described.

### 4.5. Antibacterial Activity Analysis

#### 4.5.1. Bacterial Strains Used

The bacteria were procured from Microbial Type Culture Collection and Gene Bank (MTCC), Chandigarh and maintained under standard conditions as prescribed by Bonnet, et al. [79]. The bacterial strains used include *Escherichia coli*, *Pseudomonas aeruginosa*, *Staphylococcus aureus*, *Bacillus cereus*, *Streptococcus pyogenes* and *Salmonella enterica*.

#### 4.5.2. Disc Diffusion Method

Initially, the bacteria cultured were completed in Luria-Bertani broth; for the antibacterial study, the inoculum of the bacteria was made on a Mueller Hinton Agar (MHA) agar plate (Himedia, Mumbai, Maharashtra, India) at a thickness of 5 mm. Later, a filter paper disc (8 mm in diameter) containing the leaf essential oil of *C. malabatrum* (10 μL) was placed in the agar plate at a distance of 50 mm. At the end of 24 h, the formation of the growth inhibition zone was estimated [80].

#### 4.5.3. Minimum Inhibitory Concentration (MIC)

The determination of the MIC value was made according to the methods described by Campana, et al. [81]. Before beginning, the density of the inoculum was spectrophotometrically set to 5 × 10^5^ CFU/mL. From this, about 50 µL was transferred to individual wells of a 96-well plate containing different concentrations of *C. malabatrum* essential oil. Later, 2,3,5-triphenyltetrazolium chloride (10 µL) was added to each well; the pink color of the 2,3,5-triphenyltetrazolium chloride was lost in the absence of bacterial growth. The MIC value was considered as the lowest concentration without a detectable pink color.

### 4.6. Statistical Analysis 

The results are presented as the mean± standard deviation value of three independent experiments. The statistical analysis comparison was made between the standard compounds used, linalool and essential oil by a one-way analysis of variance using GraphPad prism ver. 7.0 (San Diego, CA, USA).

## 5. Conclusions

The present study confirms the pharmacological potential of the *Cinnamomum malabatrum*, an endemic plant of Western Ghats, India. *C. malabatrum* leaf essential oil was found to have significant radical quenching abilities against different free radical sources. Likewise, the essential oil was also capable of inhibiting enzymes associated with diabetes and associated secondary complications. The strong antibacterial potential for the *C. malabatrum* essential oil was observed for both Gram-positive and Gram-negative bacteria. Hence, based on the signification of the results, the *C. malabatrum* essential oil may be a useful pharmacological agent.

## Figures and Tables

**Table 1:** Predominant compounds of *C. malabatrum* leaf essential oil (the complete list is given in Supplementary Table S1).

Retention Time	Component	Percentage Composition
13.02	Linalool	38.26 ± 0.41
11.24	Cinnamaldehyde	12.01 ± 0.54
14.34	Caryophyllene	11.43 ± 0.52
21.59	Benzyl Benzoate	9.60 ± 0.05
16.43	Eugenol	8.75 ± 0.23
15.06	Humulene	5.32 ± 0.12

**Table 2:** Radical quenching abilities of the essential oil extracted from *C. malabatrum* leaves. The values expressed are half-maximal inhibition concentration-IC_50_ (µg/mL).

	CMEO	Linalool	Ascorbic Acid
DPPH radical scavenging	21.50 ± 0.17	35.22 ± 0.11	8.13 ± 0.09
ABTS radical scavenging	36.91 ± 0.41	40.01 ± 1.33	12.82 ± 0.40
H_2_O_2_ radical scavenging	42.77 ± 0.34	38.09 ± 2.45	19.11 ± 0.26
Ferric-reducing potential	12.38 ± 0.11	35.93 ± 0.24	15.38 ± 0.66
Lipid peroxidation inhibition	85.83 ± 0.47	78.49 ± 3.07	63.02 ± 0.33

(Statistical comparison has been detailed in Supplementary Table S2).

**Table 3:** Enzyme-inhibitory abilities (IC_50_ in µg/mL) of *C. malabatrum* leaf essential oil.

Enzyme Inhibition	CMEO	Linalool	Ascorbic Acid
α-Amylase	74.19 ± 1.55	62.34 ± 2.91	45.17 ± 2.36
*α*-Glucosidase	47.07 ± 3.14	30.93 ± 3.41	36.03 ± 1.98
Aldose reductase	82.90 ± 0.67	59.04 ± 2.26	28.70 ± 2.14
Sorbitol dehydrogenase	98.61 ± 3.18	88.37 ± 3.75	60.09 ± 1.32

(Statistical comparison has been detailed in Supplementary Table S3).

**Table 4:** Antibacterial properties of *C. malabatrum* by disc diffusion method.

Bacteria	Zone of Inhibition (mm)		
	CMEO	Linalool	GM
*Staphylococcus aureus*	16.2 ± 0.3	18.1 ± 0.2	18.5 ± 0.5
*Bacillus cereus*	14.8 ± 0.4	17.6 ± 0.3	21.3 ± 0.5
*Streptococcus pyogenes*	16.7 ± 0.3	17.9 ± 0.1	19.2 ± 0.7
*Escherichia coli*	18.1 ± 0.2	17.8 ± 0.1	21.3 ± 0.3
*Pseudomonas aeruginosa*	20.8 ± 0.5	19.3 ± 0.2	21.6 ± 0.4
*Salmonella enterica*	17.4 ± 0.2	16.8 ± 0.3	19.9 ± 0.3

(Statistical comparison has been detailed in Supplementary Table S4).

**Table 5:** Minimum inhibitory concentrations (mg/mL) of *C. malabatrum* essential oil and antibiotic gentamicin.

Bacteria	MIC Value		
	CMEO	Linalool	GM
*Staphylococcus aureus*	1.25 ± 0.05	0.325 ± 0.00	0.325 ± 0.00
*Bacillus cereus*	0.75 ± 0.05	0.625 ± 0.10	0.325 ± 0.00
*Streptococcus pyogenes*	0.625 ± 0.10	0.325 ± 0.00	0.167 ± 0.00
*Escherichia coli*	1.00 ± 0.10	0.625 ± 0.05	0.325 ± 0.00
*Pseudomonas aeruginosa*	0.625 ± 0.15	0.325 ± 0.10	0.167 ± 0.00
*Salmonella enterica*	0.625 ± 0.05	0.325 ± 0.00	0.167 ± 0.00

(Statistical comparison has been detailed in Supplementary Table S5).

## Data Availability

All the data are included in the manuscript.

## Supplementary Materials

The following supporting information can be downloaded at: https://www.mdpi.com/article/10.3390/antibiotics12050940/s1. Table S1. Chemical composition of *C. malabatrum* leaf essential oil. Table S2. Statistical comparison of the antioxidant activities between the CMEO, linalool and ascorbic acid. Table S3. Statistical comparison of the enzyme-inhibitory potentials between the CMEO, linalool and ascorbic acid. Table S4. Statistical comparison of the antibacterial activity in terms of disc diffusion assay between the CMEO, linalool and gentamicin. Table S5. Statistical comparison of the antibacterial activity in terms of MIC value between the CMEO, linalool and gentamicin.

## References

[1] Kumar K.M., Asish G.R., Sabu M., Balachandran I. (2013). Significance of gingers (Zingiberaceae) in Indian System of Medicine—Ayurveda: An overview. Anc. Sci. Life.

[2] Amiri M.S., Joharchi M.R. (2013). Ethnobotanical investigation of traditional medicinal plants commercialized in the markets of Mashhad, Iran. Avicenna J. Phytomed..

[3] Chen S.L., Yu H., Luo H.M., Wu Q., Li C.F., Steinmetz A. (2016). Conservation and sustainable use of medicinal plants: Problems, progress, and prospects. Chin. Med..

[4] Saldanha L.G., Dwyer J.T., Betz J.M. (2016). Culinary Spice Plants in Dietary Supplement Products and Tested in Clinical Trials. Adv. Nutr..

[5] Dini I., Laneri S. (2021). Spices, Condiments, Extra Virgin Olive Oil and Aromas as Not Only Flavorings, but Precious Allies for Our Wellbeing. Antioxidants.

[6] Patwardhan B., Warude D., Pushpangadan P., Bhatt N. (2005). Ayurveda and traditional Chinese medicine: A comparative overview. Evid. Based Complement. Altern. Med. Ecam.

[7] Wu L., Chen W., Wang Z. (2021). Traditional Indian medicine in China: The status quo of recognition, development and research. J. Ethnopharmacol..

[8] Khalil M., Hayek S., Khalil N., Serale N., Vergani L., Calasso M., De Angelis M., Portincasa P. (2021). Role of Sumac (*Rhus coriaria* L.) in the management of metabolic syndrome and related disorders: Focus on NAFLD-atherosclerosis interplay. J. Funct. Foods.

[9] Kunnumakkara A.B., Sailo B.L., Banik K., Harsha C., Prasad S., Gupta S.C., Bharti A.C., Aggarwal B.B. (2018). Chronic diseases, inflammation, and spices: How are they linked?. J. Transl. Med..

[10] de Oliveira D.P., Braga F.C., Teixeira M.M., Gopi S., Amalraj A., Kunnumakkara A., Thomas S. (2021). Medicinal plants and their potential use in the treatment of rheumatic diseases. Inflammation and Natural Products.

[11] Kuete V., Kuete V. (2017). Chapter 13—Other Health Benefits of African Medicinal Spices and Vegetables. Medicinal Spices and Vegetables from Africa.

[12] Wang J., Su B., Jiang H., Cui N., Yu Z., Yang Y., Sun Y. (2020). Traditional uses, phytochemistry and pharmacological activities of the genus *Cinnamomum* (*Lauraceae*): A review. Fitoterapia.

[13] Cardoso-Ugarte G.A., López-Malo A., Sosa-Morales M.E., Preedy V.R. (2016). Chapter 38—Cinnamon (*Cinnamomum zeylanicum*) Essential Oils. Essential Oils in Food Preservation, Flavor and Safety.

[14] de Lima L.B., Viturino da Silva W.A., Silva S.L., Felipe Dos Santos E.C., Barbosa Machado J.C., Procopio T.F., de Moura M.C., Napoleao T.H., Assuncao Ferreira M.R., Soares L.A.L. (2022). Chemical and antibacterial analysis of *Cinnamomum verum* leaves extract and fractions against multidrug resistant bacteria. Nat. Prod. Res..

[15] Assaran A.H., Beheshti F., Marefati N., Rashidi R., Hosseini M., Bibak B., Shakeri F. (2022). Effect of hydro-alcoholic extract of *Cinnamomum zeylanicum* on nitric oxide metabolites in brain tissues following seizures induced by pentylenetetrazole in mice. Avicenna J. Phytomed..

[16] Sandamali J.A.N., Hewawasam R.P., Jayatilaka K., Mudduwa L.K.B. (2021). *Cinnamomum zeylanicum* Blume (*Ceylon cinnamon*) bark extract attenuates doxorubicin induced cardiotoxicity in Wistar rats. Saudi Pharm. J. SPJ Off. Publ. Saudi Pharm. Soc..

[17] Atsamo A.D., Lontsie Songmene A., Metchi Donfack M.F., Ngouateu O.B., Nguelefack T.B., Dimo T. (2021). Aqueous Extract from *Cinnamomum zeylanicum* (*Lauraceae*) Stem Bark Ameliorates Gentamicin-Induced Nephrotoxicity in Rats by Modulating Oxidative Stress and Inflammatory Markers. Evid.-Based Complement. Altern. Med. Ecam.

[18] Sayad-Fathi S., Zaminy A., Babaei P., Yousefbeyk F., Azizi N., Nasiri E. (2020). The methanolic extract of *Cinnamomum zeylanicum* bark improves formaldehyde-induced neurotoxicity through reduction of phospho-tau (Thr231), inflammation, and apoptosis. EXCLI J..

[19] Aryanezhad M., Abdi M., Amini S., Hassanzadeh K., Valadbeigi E., Rahimi K., Izadpanah E., Moloudi M.R. (2021). *Cinnamomum zeylanicum* extract has antidepressant-like effects by increasing brain-derived neurotrophic factor (BDNF) and its receptor in prefrontal cortex of rats. Avicenna J. Phytomed..

[20] Aggarwal S., Bhadana K., Singh B., Rawat M., Mohammad T., Al-Keridis L.A., Alshammari N., Hassan M.I., Das S.N. (2022). *Cinnamomum zeylanicum* Extract and its Bioactive Component Cinnamaldehyde Show Anti-Tumor Effects via Inhibition of Multiple Cellular Pathways. Front. Pharmacol..

[21] Alanazi A.D., Almohammed H.I. (2022). Therapeutic Potential and Safety of the *Cinnamomum zeylanicum* Methanolic Extract against Chronic *Toxoplasma gondii* Infection in Mice. Front. Cell. Infect. Microbiol..

[22] Lee J.H., Kwak H.J., Shin D., Seo H.J., Park S.J., Hong B.H., Shin M.S., Kim S.H., Kang K.S. (2022). Mitigation of Gastric Damage Using Cinnamomum cassia Extract: Network Pharmacological Analysis of Active Compounds and Protection Effects in Rats. Plants.

[23] Susilowati R., Setiawan A.M., Zahroh A.F., Ashari Z.N., Iffiyana A., Hertanto R., Basyarudin M., Hartiningsih I., Ismail M. (2022). Hepatoprotection of *Cinnamomum burmannii* ethanolic extract against high-fat and cholesterol diet in Sprague-Dawley rats (*Rattus norvegicus*). Vet. World.

[24] Panjaitan C.C., Widyarman A.S., Amtha R., Astoeti T.E. (2022). Antimicrobial and Antibiofilm Activity of Cinnamon (*Cinnamomum burmanii*) Extract on Periodontal Pathogens-An in vitro study. Eur. J. Dent..

[25] Yu H., Ren X., Yang F., Xie Y., Guo Y., Cheng Y., Yao W. (2022). Antimicrobial and anti-dust mite efficacy of *Cinnamomum camphora chvar*. Borneol essential oil using pilot-plant neutral cellulase-assisted steam distillation. Lett. Appl. Microbiol..

[26] Mujawah A.A.H., Abdallah E.M., Alshoumar S.A., Alfarraj M.I., Alajel S.M.I., Alharbi A.L., Alsalman S.A., Alhumaydhi F.A. (2022). GC-MS and in vitro antibacterial potential of *Cinnamomum camphora* essential oil against some clinical antibiotic-resistant bacterial isolates. Eur. Rev. Med. Pharmacol. Sci..

[27] Xu Y., Qin J., Wang P., Li Q., Yu S., Zhang Y., Wang Y. (2020). Chemical composition and larvicidal activities of essential oil of *Cinnamomum camphora* (L.) leaf against Anopheles stephensi. Rev. Soc. Bras. Med. Trop..

[28] Xiao S., Yu H., Xie Y., Guo Y., Fan J., Yao W. (2021). The anti-inflammatory potential of *Cinnamomum camphora* (L.) J.Presl essential oil in vitro and in vivo. J. Ethnopharmacol..

[29] Chen J., Tang C., Zhou Y., Zhang R., Ye S., Zhao Z., Lin L., Yang D. (2020). Anti-Inflammatory Property of the Essential Oil from *Cinnamomum camphora* (Linn.) Presl Leaves and the Evaluation of Its Underlying Mechanism by Using Metabolomics Analysis. Molecules.

[30] Xiao S., Yu H., Xie Y., Guo Y., Fan J., Yao W. (2021). Evaluation of the analgesic potential and safety of *Cinnamomum camphora chvar*. Borneol essential oil. Bioengineered.

[31] Tang Y., Lv X., Liu Y., Cui D., Wu Y. (2022). Metabonomics Study in Mice with Learning and Memory Impairment on the Intervention of Essential Oil Extracted from *Cinnamomum camphora chvar*. Borneol. Front. Pharmacol..

[32] Kallel I., Hadrich B., Gargouri B., Chaabane A., Lassoued S., Gdoura R., Bayoudh A., Ben Messaoud E. (2019). Optimization of Cinnamon (*Cinnamomum zeylanicum* Blume) Essential Oil Extraction: Evaluation of Antioxidant and Antiproliferative Effects. Evid. Based Complement. Altern. Med. Ecam.

[33] Alizadeh Behbahani B., Falah F., Lavi Arab F., Vasiee M., Tabatabaee Yazdi F. (2020). Chemical Composition and Antioxidant, Antimicrobial, and Antiproliferative Activities of *Cinnamomum zeylanicum* Bark Essential Oil. Evid. Based Complement. Altern. Med. Ecam.

[34] Bellassoued K., Ghrab F., Hamed H., Kallel R., van Pelt J., Lahyani A., Ayadi F.M., El Feki A. (2019). Protective effect of essential oil of *Cinnamomum verum* bark on hepatic and renal toxicity induced by carbon tetrachloride in rats. Appl. Physiol. Nutr. Metab. Physiol. Appl. Nutr. Metab..

[35] Sriramavaratharajan V., Murugan R. (2019). Evaluation of chemical composition, antioxidant and anti-hyperglycemic activities of the essential oil based nanoemulsions of *Cinnamomum litseifolium*. Nat. Prod. Res..

[36] Azab S.S., Abdel Jaleel G.A., Eldahshan O.A. (2017). Anti-inflammatory and gastroprotective potential of leaf essential oil of *Cinnamomum glanduliferum* in ethanol-induced rat experimental gastritis. Pharm. Biol..

[37] Cheng B.H., Sheen L.Y., Chang S.T. (2018). Hypolipidemic effects of S-(+)-linalool and essential oil from *Cinnamomum osmophloeum* ct. linalool leaves in mice. J. Tradit. Complement. Med..

[38] Lee S.C., Xu W.X., Lin L.Y., Yang J.J., Liu C.T. (2013). Chemical composition and hypoglycemic and pancreas-protective effect of leaf essential oil from indigenous cinnamon (*Cinnamomum osmophloeum* Kanehira). J. Agric. Food Chem..

[39] Lee S.C., Hsu J.S., Li C.C., Chen K.M., Liu C.T. (2015). Protective effect of leaf essential oil from *Cinnamomum osmophloeum* Kanehira on endotoxin-induced intestinal injury in mice associated with suppressed local expression of molecules in the signaling pathways of TLR4 and NLRP3. PloS ONE.

[40] Leela N.K., Vipin T.M., Shafeekh K.M., Priyanka V., Rema J. (2009). Chemical composition of essential oils from aerial parts of *Cinnamomum malabatrum* (Burman f.) Bercht & Presl. Flavour Fragr. J..

[41] Sriramavaratharajan V., Murugan R. (2020). Chemical Profiling of the Leaf Essential Oils of Cinnamomum Species Used as a Spice in Southern India. J. Biol. Act. Prod. Nat..

[42] Kaur N., Kumar V., Nayak S.K., Wadhwa P., Kaur P., Sahu S.K. (2021). Alpha-amylase as molecular target for treatment of diabetes mellitus: A comprehensive review. Chem. Biol. Drug Des..

[43] Alqahtani A.S., Hidayathulla S., Rehman M.T., ElGamal A.A., Al-Massarani S., Razmovski-Naumovski V., Alqahtani M.S., El Dib R.A., AlAjmi M.F. (2019). Alpha-Amylase and Alpha-Glucosidase Enzyme Inhibition and Antioxidant Potential of 3-Oxolupenal and Katononic Acid Isolated from *Nuxia oppositifolia*. Biomolecules.

[44] Santoso M., Ong L.L., Aijijiyah N.P., Wati F.A., Azminah A., Annuur R.M., Fadlan A., Judeh Z.M.A. (2022). Synthesis, α-glucosidase inhibition, α-amylase inhibition, and molecular docking studies of 3,3-di(indolyl)indolin-2-ones. Heliyon.

[45] Dirir A.M., Daou M., Yousef A.F., Yousef L.F. (2022). A review of alpha-glucosidase inhibitors from plants as potential candidates for the treatment of type-2 diabetes. Phytochem. Rev..

[46] Hashimoto Y., Yamagishi S., Mizukami H., Yabe-Nishimura C., Lim S.W., Kwon H.M., Yagihashi S. (2011). Polyol pathway and diabetic nephropathy revisited: Early tubular cell changes and glomerulopathy in diabetic mice overexpressing human aldose reductase. J. Diabetes Investig..

[47] Jia G., Hill M.A., Sowers J.R. (2018). Diabetic Cardiomyopathy. Circ. Res..

[48] Mara L., Oates P.J., Duh E.J. (2008). The Polyol Pathway and Diabetic Retinopathy. Diabetic Retinopathy.

[49] Pizzino G., Irrera N., Cucinotta M., Pallio G., Mannino F., Arcoraci V., Squadrito F., Altavilla D., Bitto A. (2017). Oxidative Stress: Harms and Benefits for Human Health. Oxidative Med. Cell. Longev..

[50] Lobo V., Patil A., Phatak A., Chandra N. (2010). Free radicals, antioxidants and functional foods: Impact on human health. Pharmacogn. Rev..

[51] Sharifi-Rad M., Anil Kumar N.V., Zucca P., Varoni E.M., Dini L., Panzarini E., Rajkovic J., Tsouh Fokou P.V., Azzini E., Peluso I. (2020). Lifestyle, Oxidative Stress, and Antioxidants: Back and Forth in the Pathophysiology of Chronic Diseases. Front. Physiol..

[52] Seol G.H., Kang P., Lee H.S. (2016). Antioxidant activity of linalool in patients with carpal tunnel syndrome. BMC Neurol..

[53] Dahham S.S., Tabana Y.M., Iqbal M.A., Ahamed M.B., Ezzat M.O., Majid A.S., Majid A.M. (2015). The Anticancer, Antioxidant and Antimicrobial Properties of the Sesquiterpene β-Caryophyllene from the Essential Oil of *Aquilaria crassna*. Molecules.

[54] Subash-Babu P., Alshatwi A.A., Ignacimuthu S. (2014). Beneficial Antioxidative and Antiperoxidative Effect of Cinnamaldehyde Protect Streptozotocin-Induced Pancreatic β-Cells Damage in Wistar Rats. Biomol. Ther..

[55] Suryanti V., Wibowo F.R., Khotijah S., Andalucki N. (2018). Antioxidant Activities of Cinnamaldehyde Derivatives. IOP Conf. Ser. Mater. Sci. Eng..

[56] Cade W.T. (2008). Diabetes-related microvascular and macrovascular diseases in the physical therapy setting. Phys. Ther..

[57] Chawla A., Chawla R., Jaggi S. (2016). Microvasular and macrovascular complications in diabetes mellitus: Distinct or continuum?. Indian J. Endocrinol. Metab..

[58] Shahin D.H.H., Sultana R., Farooq J., Taj T., Khaiser U.F., Alanazi N.S.A., Alshammari M.K., Alshammari M.N., Alsubaie F.H., Asdaq S.M.B. (2022). Insights into the Uses of Traditional Plants for Diabetes Nephropathy: A Review. Curr. Issues Mol. Biol..

[59] Lorenzi M. (2007). The polyol pathway as a mechanism for diabetic retinopathy: Attractive, elusive, and resilient. Exp. Diabetes Res..

[60] Braz V.S., Melchior K., Moreira C.G. (2020). *Escherichia coli* as a Multifaceted Pathogenic and Versatile Bacterium. Front. Cell. Infect. Microbiol..

[61] Gellatly S.L., Hancock R.E.W. (2013). *Pseudomonas aeruginosa*: New insights into pathogenesis and host defenses. Pathog. Dis..

[62] Ibrahim J., Eisen J.A., Jospin G., Coil D.A., Khazen G., Tokajian S. (2016). Genome Analysis of *Streptococcus pyogenes* Associated with Pharyngitis and Skin Infections. PloS ONE.

[63] Tong S.Y., Davis J.S., Eichenberger E., Holland T.L., Fowler V.G. (2015). Staphylococcus aureus infections: Epidemiology, pathophysiology, clinical manifestations, and management. Clin. Microbiol. Rev..

[64] Messelhäußer U., Ehling-Schulz M. (2018). *Bacillus cereus*—A Multifaceted Opportunistic Pathogen. Curr. Clin. Microbiol. Rep..

[65] Bottone E.J. (2010). Bacillus cereus, a volatile human pathogen. Clin. Microbiol. Rev..

[66] Guimarães A.C., Meireles L.M., Lemos M.F., Guimarães M.C.C., Endringer D.C., Fronza M., Scherer R. (2019). Antibacterial Activity of Terpenes and Terpenoids Present in Essential Oils. Molecules.

[67] Berthold-Pluta A., Stasiak-Różańska L., Pluta A., Garbowska M. (2019). Antibacterial activities of plant-derived compounds and essential oils against Cronobacter strains. Eur. Food Res. Technol..

[68] Sawicki R., Golus J., Przekora A., Ludwiczuk A., Sieniawska E., Ginalska G. (2018). Antimycobacterial Activity of Cinnamaldehyde in a *Mycobacterium tuberculosis* (H37Ra) Model. Molecules.

[69] Visakh N.U., Pathrose B., Narayanankutty A., Alfarhan A., Ramesh V. (2022). Utilization of Pomelo (*Citrus maxima*) Peel Waste into Bioactive Essential Oils: Chemical Composition and Insecticidal Properties. Insects.

[70] Kamal F.Z., Stanciu G.D., Lefter R., Cotea V.V., Niculaua M., Ababei D.C., Ciobica A., Ech-Chahad A. (2022). Chemical Composition and Antioxidant Activity of *Ammi visnaga* L. Essential Oil. Antioxidants.

[71] Li Y., Liu S., Zhao C., Zhang Z., Nie D., Tang W. (2022). The Chemical Composition and Antibacterial and Antioxidant Activities of Five Citrus Essential Oils. Molecules.

[72] Munteanu I.G., Apetrei C. (2021). Analytical Methods Used in Determining Antioxidant Activity: A Review. Int. J. Mol. Sci..

[73] He T., Li X., Wang X., Xu X., Yan X., Li X., Sun S., Dong Y., Ren X., Liu X. (2020). Chemical composition and anti-oxidant potential on essential oils of *Thymus quinquecostatus* Celak. from Loess Plateau in China, regulating Nrf2/Keap1 signaling pathway in zebrafish. Sci. Rep..

[74] Okoh S.O., Asekun O.T., Familoni O.B., Afolayan A.J. (2014). Antioxidant and Free Radical Scavenging Capacity of Seed and Shell Essential Oils Extracted from *Abrus precatorius* (L). Antioxidants.

[75] Quan N.V., Xuan T.D., Tran H.-D., Thuy N.T.D., Trang L.T., Huong C.T., Andriana Y., Tuyen P.T. (2019). Antioxidant, α-Amylase and α-Glucosidase Inhibitory Activities and Potential Constituents of *Canarium tramdenum* Bark. Molecules.

[76] Assefa S.T., Yang E.-Y., Chae S.-Y., Song M., Lee J., Cho M.-C., Jang S. (2020). Alpha Glucosidase Inhibitory Activities of Plants with Focus on Common Vegetables. Plants.

[77] Ali M.Y., Zaib S., Jannat S., Khan I., Rahman M.M., Park S.K., Chang M.S. (2022). Inhibition of Aldose Reductase by Ginsenoside Derivatives via a Specific Structure Activity Relationship with Kinetics Mechanism and Molecular Docking Study. Molecules.

[78] Kazeem M.I., Adeyemi A.A., Adenowo A.F., Akinsanya M.A. (2020). *Carica papaya* Linn. fruit extract inhibited the activities of aldose reductase and sorbitol dehydrogenase: Possible mechanism for amelioration of diabetic complications. Future J. Pharm. Sci..

[79] Bonnet M., Lagier J.C., Raoult D., Khelaifia S. (2019). Bacterial culture through selective and non-selective conditions: The evolution of culture media in clinical microbiology. New Microbes New Infect.

[80] Walia S., Mukhia S., Bhatt V., Kumar R., Kumar R. (2020). Variability in chemical composition and antimicrobial activity of *Tagetes minuta* L. essential oil collected from different locations of Himalaya. Ind. Crops Prod..

[81] Campana R., Tiboni M., Maggi F., Cappellacci L., Cianfaglione K., Morshedloo M.R., Frangipani E., Casettari L. (2022). Comparative Analysis of the Antimicrobial Activity of Essential Oils and Their Formulated Microemulsions against Foodborne Pathogens and Spoilage Bacteria. Antibiotics.

