# Supplementary material for: Chemical Composition of the Cinnamomum malabatrum Leaf Essential Oil and Analysis of Its Antioxidant, Enzyme Inhibitory and Antibacterial Activities
Source: Antibiotics (Basel). 2023 May 22;12(5):940. doi: 10.3390/antibiotics12050940 (PMC10215355; doi:10.3390/antibiotics12050940)
Supplement: Supplementary file 1 [file antibiotics-12-00940-s001.zip › antibiotics-2042592-supplementary.pdf]

Supplementary Table S1. Chemical composition of *C. malabatrum* leaf essential oil.

Peak no.	RT[a]	Component	RI[b]	RI[c]	%RA [d]
1	5.99	Benzaldehyde	976	966	1.50±0.04
2	8.14	Linalyl acetate	1174	1104	1.36±0.07
3	11.24	Cinnamaldehyde	1277	1282	12.01±0.54
4	13.02	Linalool	1342	1371	38.26±0.41
5	13.82	Methyleugenol	1410	1410	3.40±0.67
6	14.34	Caryophyllene	1433	1435	11.43±0.52
7	14.69	Cinnamyl acetate	1445	1451	3.26±0.08
8	15.06	Humulene	1464	1469	5.32±0.12
9	16.43	Eugenol	1482	1533	8.75±0.23
10	17.85	Ledene oxide	1612	1599	3.09±0.78
11	21.59	Benzyl Benzoate	1703	1677	9.60±0.05

[a] Retention time; [b] Library retention index; [c] Calculated retention index; [d] Relative area.

Supplementary Table S2. Statistical comparison of the antioxidant activities between the CMEO, linalool and ascorbic acid.

Tukey's multiple comparisons test	Mean Difference	95.00% CI of difference	Adjusted P Value
**DPPH**			
CMEO vs. Linalool	-13.72	-15.26 to -12.18	<0.0001
CMEO vs. Ascorbic acid	13.37	11.83 to 14.91	<0.0001
Linalool vs. Ascorbic acid	27.09	25.55 to 28.63	<0.0001
**ABTS**			
CMEO vs. Linalool	-3.1	-4.637 to -1.563	<0.0001
CMEO vs. Ascorbic acid	24.09	22.55 to 25.63	<0.0001
Linalool vs. Ascorbic acid	27.19	25.65 to 28.73	<0.0001
**H2O2**			
CMEO vs. Linalool	4.68	3.143 to 6.217	<0.0001
CMEO vs. Ascorbic acid	23.66	22.12 to 25.2	<0.0001
Linalool vs. Ascorbic acid	18.98	17.44 to 20.52	<0.0001
**FRAP**			
CMEO vs. Linalool	-23.55	-25.09 to -22.01	<0.0001
CMEO vs. Ascorbic acid	-3	-4.537 to -1.463	<0.0001
Linalool vs. Ascorbic acid	20.55	19.01 to 22.09	<0.0001
**LPO**			
CMEO vs. Linalool	7.34	5.803 to 8.877	<0.0001
CMEO vs. Ascorbic acid	22.81	21.27 to 24.35	<0.0001
Linalool vs. Ascorbic acid	15.47	13.93 to 17.01	<0.0001

Supplementary Table S3. Statistical comparison of the enzyme-inhibitory potentials between the CMEO, linalool and ascorbic acid.

Tukey's multiple comparisons test	Mean Diff.	95.00% CI of diff.	Adjusted P Value
**a-Amylase**			
CMEO vs. Linalool	11.85	8.315 to 15.39	<0.0001
CMEO vs. Ascorbic acid	29.02	25.48 to 32.56	<0.0001
Linalool vs. Ascorbic acid	17.17	13.63 to 20.71	<0.0001
**a-Glucosidase**			
CMEO vs. Linalool	16.14	12.6 to 19.68	<0.0001
CMEO vs. Ascorbic acid	11.04	7.505 to 14.58	<0.0001
Linalool vs. Ascorbic acid	-5.1	-8.635 to -1.565	0.0028
**Aldose reductase**			
CMEO vs. Linalool	23.86	20.32 to 27.4	<0.0001
CMEO vs. Ascorbic acid	54.2	50.66 to 57.74	<0.0001
Linalool vs. Ascorbic acid	30.34	26.8 to 33.88	<0.0001
**Sorbitol dehydrogenase**			
CMEO vs. Linalool	10.24	6.705 to 13.78	<0.0001
CMEO vs. Ascorbic acid	38.52	34.98 to 42.06	<0.0001
Linalool vs. Ascorbic acid	28.28	24.74 to 31.82	<0.0001

Supplementary Table S4. Statistical comparison of the antibacterial activity in terms of disc diffusion assay between the CMEO, linalool and gentamicin.

**Tukey's multiple comparisons test**	**Mean Diff.**	**95.00% CI of diff.**	**Adjusted P Value**
Escherichia coli			
CMEO vs. Linalool	0.3	-0.1897 to 0.7897	0.3150
CMEO vs. Genamicin	-3.2	-3.69 to -2.71	<0.0001
Linalool vs. Genamicin	-3.5	-3.99 to -3.01	<0.0001
Pseudomonas aeruginosa			
CMEO vs. Linalool	1.5	1.01 to 1.99	<0.0001
CMEO vs. Genamicin	-0.8	-1.29 to -0.3103	0.0006
Linalool vs. Genamicin	-2.3	-2.79 to -1.81	<0.0001
Staphylococcus aureus			
CMEO vs. Linalool	-1.9	-2.39 to -1.41	<0.0001
CMEO vs. Genamicin	-2.3	-2.79 to -1.81	<0.0001
Linalool vs. Genamicin	-0.4	-0.8897 to 0.08968	0.1317
Bacillus cereus			
CMEO vs. Linalool	-2.8	-3.29 to -2.31	<0.0001
CMEO vs. Genamicin	-6.5	-6.99 to -6.01	<0.0001
Linalool vs. Genamicin	-3.7	-4.19 to -3.21	<0.0001
Streptococcus pyogenes			
CMEO vs. Linalool	-1.2	-1.69 to -0.7103	<0.0001
CMEO vs. Genamicin	-2.5	-2.99 to -2.01	<0.0001
Linalool vs. Genamicin	-1.3	-1.79 to -0.8103	<0.0001
Salmonella enterica			
CMEO vs. Linalool	0.6	0.1103 to 1.09	0.0122
CMEO vs. Genamicin	-2.5	-2.99 to -2.01	<0.0001
Linalool vs. Genamicin	-3.1	-3.59 to -2.61	<0.0001

Supplementary Table S5. Statistical comparison of the antibacterial activity in terms of MIC value between the CMEO, linalool and gentamicin.

**Tukey's multiple comparisons test**	**Mean Difference**	**95.00% CI of difference**	**Adjusted P Value**
Escherichia coli			
CMEO vs. Linalool	0.375	0.2877 to 0.4623	<0.0001
CMEO vs. Genamicin	0.675	0.5877 to 0.7623	<0.0001
Linalool vs. Genamicin	0.3	0.2127 to 0.3873	<0.0001
Pseudomonas aeruginosa			
CMEO vs. Linalool	0.3	0.2127 to 0.3873	<0.0001
CMEO vs. Genamicin	0.458	0.3707 to 0.5453	<0.0001
Linalool vs. Genamicin	0.158	0.07068 to 0.2453	0.0001
Staphylococcus aureus			
CMEO vs. Linalool	0.925	0.8377 to 1.012	<0.0001
CMEO vs. Genamicin	0.925	0.8377 to 1.012	<0.0001
Linalool vs. Genamicin	0	-0.08732 to 0.08732	>0.9999
Bacillus cereus			
CMEO vs. Linalool	0.125	0.03768 to 0.2123	0.0028
CMEO vs. Genamicin	0.425	0.3377 to 0.5123	<0.0001
Linalool vs. Genamicin	0.3	0.2127 to 0.3873	<0.0001
Streptococcus pyogenes			
CMEO vs. Linalool	0.3	0.2127 to 0.3873	<0.0001
CMEO vs. Genamicin	0.458	0.3707 to 0.5453	<0.0001
Linalool vs. Genamicin	0.158	0.07068 to 0.2453	0.0001
Salmonella enterica			
CMEO vs. Linalool	0.3	0.2127 to 0.3873	<0.0001
CMEO vs. Genamicin	0.458	0.3707 to 0.5453	<0.0001
Linalool vs. Genamicin	0.158	0.07068 to 0.2453	0.0001
